# Personal

**Published:** 1884-06

**Authors:** 


### PERSONAL.

It will be a gratification to the many admirers of Dr. Miller to know that he sails from Bremen by the steamer Eider, June 11th, for a brief visit to his native land, and will be due in New York about June 20th. He will remain in this country only about a month.

It will give the readers of the INDEPENDENT PRACTITIONER yet greater pleasure to know that Dr. Miller is now permanently connected with this journal as one of its publishing company, and that henceforth his interest in it will be personal as well as professional. As for the other members of the company, the heartiness with which they welcome him to an honorable position among the dentists of America, where he most desires to feel that he has a place, and the satisfaction with which they greet him as a responsible co-laborer, can be readily appreciated by readers of the journal, and will be made manifest to him when he arrives among us.

